# A service evaluation of Early Positive Approaches to Support (E-PAtS): Comparing online versus in-person group outcomes

**DOI:** 10.1192/bjb.2025.10181

**Published:** 2026-08

**Authors:** Hannah Newton, Emma Johnston, Nick Gore, Caitlin A. Williams, Vaso Totsika, Jill Bradshaw

**Affiliations:** 1 Llygad Early Years Experience Team, Cardiff and Vale University Health Board, Cardiff, UK; 2 Intellectual Disabilities Research Institute (IDRIS), University of Birmingham, Birmingham, UK; 3 Division of Psychiatry, University College London, London, UK; 4 School of Psychology, University of Birmingham, Birmingham, UK; 5 Tavistock & Portman NHS Foundation Trust, London, UK

**Keywords:** Intellectual disability, patients and service users, service development, psychosocial interventions, mental well-being

## Abstract

**Aims and method:**

Early Positive Approaches to Support (E-PAtS) is a co-produced group intervention supporting family carers of children (0–5 years) with additional developmental needs. This study compared online (*n* = 10) and in-person (*n* = 11) groups to investigate whether mode of delivery was associated with different outcomes. Participants were 98 family carers reporting on their mental well-being, self-efficacy, child symptoms and knowledge pre and post intervention. Generalised estimating equations compared outcomes between groups, controlling for group cluster effects.

**Results:**

Mental well-being improved significantly across both groups (*d* = 0.47, 95% CI: 0.30, 0.63), as did an in-session measure concerning mechanisms of change (*d* = 1.28, 95% CI: 0.97, 1.59) and all other assessed outcomes. There were no significant differences in measured outcomes between online and in-person groups.

**Clinical implications:**

Establishing the equivalence of in-person with online groups is an important first step for improving service reach and support access for families of children with additional developmental needs.

Early Positive Approaches to Support (E-PAtS) is a co-produced group programme for families of young children with additional developmental needs, based on a developmental systems model.^1^ E-PAtS consists of eight group sessions (2.5 h each), co-facilitated by a family carer and a practitioner who deliver the sessions jointly. E-PAtS is a psychoeducation programme with sessions focusing on parental well-being and access to services and proactively addressing areas of positive development for children. After taking part in E-PAtS, family carers reported feeling more confident, being more aware of the need for self-care, increases in their child’s adaptive skills and reductions in behaviours that challenge.^1^ Emerging evidence suggests that E-PAtS has the potential to support family carer mental well-being,^2^ and that most family carers attend the majority of in-person sessions.

In-person attendance at programmes in general is not always easy for family carers whose lives are often very complex. Ease of accessing support is an important feature of service provision for these families.^3^ Family carers report high levels of unmet need for service support, yet under 20% access any type of intervention.^4^ Digitally enabled care is considered key for increasing access to services and reducing waiting lists, especially for specialist services for children with learning disabilities.^5^ The COVID-19 pandemic accelerated the testing of online support.^6^ Establishing that online interventions work in the same way and have the same effects as in-person models of support is important for the wider adoption of online modes of delivery. The current study compared online with in-person E-PAtS groups, as delivered to family carers of young children with additional developmental needs accessing early support services.

## Method

### Participants

All participants were family carers (i.e. adult carers including parents, adult siblings and grandparents) whose children were referred to an early support service with additional (often not yet diagnosed) learning needs. Families were referred by a health care professional, usually a health visitor, when their child scored two items below age-expected norms on the Schedule of Growing Skills assessment in two or more areas, indicating a developmental delay. Participants were then contacted, given more information about E-PAtS and offered the option to attend either in-person or online. Groups took place between November 2021 and April 2024. A total of 98 family carers were included in this study.

Demographic information was available for 59 of the 98 family carers (Table 1); of these, 56 were female and 3 were male. The average age of carers was 35 years (s.d. 5.9, range 24–53). The average age of the child for whom the family attended was 3 years (s.d. 0.9, range 2–5). Within the sample, 67% were of White ethnicity, 12% Asian, 2% Indian, 5% Black African, 6% of another Black background and 8% of mixed ethnicity.

**Table 1:** Demographic characteristics of participants receiving Early Positive Approaches to Support (*N* = 98) Table 1 long description.

Demographic characteristics	
Age (years)	
Mean (s.d.)	34.56 (6.86)
Range	24–53
Missing information	43 (43)
Gender, *n* (%)	
Female	55 (55)
Male	3 (3)
Missing information	42 (42)
Caregiver type, *n* (%)	
Primary carer	74 (74)
Secondary carer	3 (3)
Missing information	23 (23)
Ethnicity, *n* (%)	
White British	23 (23)
White other	8 (8)
African	2 (2)
Indian	1 (1)
Asian	5 (5)
Asian other	2 (2)
Black other	2 (2)
Multiple ethnic	3 (3)
Any other ethnic group	1 (1)
Missing information	53 (53)

In terms of the facilitators, demographic information was available for 5 of the 10 family carer facilitators (mean age 42.6 years, s.d. 5.4, range 37–49) and 7 of the 9 practitioner facilitators (mean age 47.0 years, s.d. 7.1, range 34–55). All facilitators described themselves as female and White British. All practitioner facilitators had a background in either psychology, early years, occupational therapy or clinical services, and held relevant undergraduate and/or postgraduate qualifications. Family carer facilitators had roles including parenting practitioners, teaching assistants and support workers, and many held qualifications in education, health or child development.

### Measures

The following four measures were used.

Enhanced well-being for family carers is considered the primary outcome for E-PAtS. The Warwick-Edinburgh Mental Well Being Scale (WEMWBS)^7^ was used to assess participants’ general mental well-being. This 14-item scale covers key aspects of psychological functioning, including autonomy, agency, curiosity, clarity of thought and positive relationships, each rated on a five-point scale from ‘none of the time’ to ‘all of the time’.

Other positive changes in psychological functioning for family carers are also targeted outcomes for E-PAtS. The Brief Parental Self-Efficacy Scale (BPSES)^8^ was used to assess participants’ belief in their ability to perform their parenting role with success. It contains five statements rated on a five-point Likert scale, from ‘strongly agree’ to ‘strongly disagree’.

The Sheffield Learning Disabilities Outcome Measure (SLDOM)^9^ was used to assess carers’ perceptions of understanding their child’s behaviour and diagnosis, confidence in managing their child, perceptions of their own parenting, impact on their wider family and confidence in engaging with other services. It includes eight items rated on a five-point Likert scale, from ‘strongly agree’ to ‘strongly disagree’, and a ‘not applicable’ category.

The Early Positive Approaches to Support Programme Evaluation tool (used as standard within E-PAtS) was also used to assess participants’ knowledge of the support available to them, their perceived ability to support their own well-being and their perceived knowledge of how to support their child’s development and behaviour. It includes ten statements rated on a five-point Likert scale, from ‘strongly agree’ to ‘strongly disagree’. This measure relates to some of the mechanisms of change associated with E-PAtS as detailed in the E-PAtS logic model.

### Procedure

Participants were given information verbally on the purpose of data collection, and were also given the opportunity to ask any questions. They were assured that all data collected would remain anonymous and be stored securely. Written informed consent was obtained from participants to support later write-up but, given that the data were collected in the context of a service evaluation through Cardiff and Vale University Health Board, the research did not require ethical review by an ethics committee. The authors assert that all procedures contributing to this work comply with the ethical standards of the relevant national and institutional committees on human experimentation, and with the Helsinki Declaration of 1975 as revised in 2013. Participants completed all measures before the first session and at the end of the final E-PAtS session (for BPSES and the E-PAtS Evaluation tool). As the intervention was offered as part of regular service provision over time, data for all measures were not available for all participants. The SLDOM and BPSES were introduced in later groups and were not completed by participants in earlier groups. Participants were emailed 3–6 months following completion of an E-PAtS group, and were asked to complete the SLDOM and WEMWBS where these were used.

### Results

Ten groups were facilitated online (mean group size 4.60, s.d. 1.27, range 3–7), while 11 took place in-person (mean group size 4.91, s.d. 2.17, range 2–9). There were no significant differences in group size between online and in-person groups (*U* = 54.50, *p* = 0.971). To examine between-group differences, generalised estimating equations (GEEs^10^) were fitted because these allow accounting for clustering within groups. GEEs were fitted for each outcome to examine whether the intervention delivery method (online versus in-person), time (pre or post intervention) or the interaction between these predictors was significant. We were unable to account for other potentially important covariates, such as caregiver gender, age or ethnicity, due to the proportion of missing demographic data. The Wald chi-square test was used to determine model significance when comparing outcomes pre versus post intervention (time) and online versus in-person (delivery method).

There was a significant time effect for each outcome: WEMWBS (Wald *χ* ^2^ = 44.32, *p* < 0.001), BPSES (Wald *χ* ^2^ = 30.31, *p* < 0.001), SLDOM (Wald *χ* ^2^ = 12.29, *p* < 0.001) and the E-PAtS Evaluation tool (Wald *χ* ^2^ = 193.37, *p* < 0.001). However, there were no significant delivery method effects for any of the outcomes: WEMWBS (Wald *χ* ^2^ = 0.96, *p* = 0.327), BPSES (Wald *χ* ^2^ = 1.40, *p* = 0.237), SLDOM (Wald *χ* ^2^ = 0.49, *p* = 0.482) and the E-PAtS Evaluation tool (Wald *χ* ^2^ = 2.26, *p* = 0.133). Additionally, there was no significant interaction between time and delivery method for any of the outcomes: WEMWBS (Wald *χ* ^2^ = 0.628, *p* = 0.428), BPSES (Wald *χ* ^2^ = 2.23, *p* = 0.136), SLDOM (Wald *χ* ^2^ = 0.20, *p* = 0.655) and the E-PAtS Evaluation tool (Wald *χ* ^2^ = 0.666, *p* = 0.415).

These findings suggest that all outcomes improved significantly from baseline to post intervention, and this increase was similar regardless of whether the family attended E-PAtS online or in-person. Tables 2 and 3 present adjusted marginal means and effect sizes for outcomes, by time and delivery method, respectively.

**Table 2:** Means and effect sizes for outcomes by pre- versus post-intervention delivery Table 2 long description.

Outcome	Pre-intervention (95% CI)	Post-intervention (95% CI)	Cohen’s *d* (95% CI)
E-PAtS programme evaluation	34.21 (33.00, 35.42)	42.23 (41.05, 43.41)	1.28 (0.97, 1.59)
Warwick-Edinburgh Mental Well Being Scale	42.41 (40.43, 44.39)	49.97 (47.06, 52.88)	0.47 (0.30, 0.63)
Brief Parental Self-Efficacy Scale	18.04 (17.11, 18.98)	22.48 (21.14, 23.81)	0.73 (0.47, 0.99)
Sheffield Learning Disability Outcome Measure	26.16 (25.08, 27.25)	29.08 (27.20, 30.97)	0.34 (0.11, 0.56)

E-PAtS, Early Positive Approaches to Support. Adjusted marginal means and effect sizes after fitting generalised estimating equations.

**Table 3:** Means and effect sizes for outcomes by online versus in-person delivery method Table 3 long description.

Outcome	Online (95% CI)	In-person (95% CI)	Cohen’s *d* (95% CI)
E-PAtS programme evaluation	39.03 (37.38, 40.67)	37.41 (36.11, 38.72)	0.25 (−0.07, 0.56)
Warwick-Edinburgh Mental Well Being Scale	47.30 (43.50, 51.10)	45.08 (42.75, 47.41)	0.16 (−0.16, 0.48)
Brief Parental Self-Efficacy Scale	20.77 (19.47, 22.06)	19.75 (18.68, 20.82)	0.23 (−0.19, 0.65)
Sheffield Learning Disability Outcome Measure	27.15 (24.87, 29.44)	28.09 (26.83, 29.34)	−0.15 (−0.54, 0.24)

E-PAtS, Early Positive Approaches to Support. Adjusted marginal means and effect sizes after fitting generalised estimating equations.

## Discussion

Given the high levels of unmet needs and challenges in accessing available support for families of children with additional developmental needs,^4^ the current study compared online with in-person E-PAtS groups. The study reports on data from a diverse sample of 98 families gathered through ongoing service evaluation. Findings suggest that both modes of delivery are associated with positive outcomes (i.e. with regard to WEMWBS, SLDOM and BPSES) and within-session mechanisms of change (i.e. the E-PAtS Evaluation tool), as predicted by the E-PAtS logic model.

Findings add to the limited evidence base currently available about the comparability of online with in-person delivery of group interventions. There is emerging evidence that group parenting interventions are equally effective when delivered online or in-person,^11,12^ but no studies to date have compared parenting groups for families of children with a learning disability.

A recent randomised trial of a group parenting intervention for parents of young children with a learning disability that moved to online delivery because of COVID-19 found that reductions in the primary outcome (children’s behaviour problems) were less substantial following the pandemic,^13^ although this may not necessarily have been due to the online format of group delivery, but rather due to the effect of the pandemic on the behaviour problems of children with a learning disability.

As a service evaluation, the current study was not purposefully designed to test effectiveness. The findings, however, contribute to the growing evidence base for E-PAtS^1,2^ and suggest that both online and face-to-face E-PAtS groups may be helpful options for families in practice. There were some limitations of the study that must be considered. First, some gaps in demographic information for participants, and a lack of information concerning certain factors (e.g. socioeconomic status), reduced our ability to control for contextual factors when exploring between-group differences. Additionally, in this study we did not collect qualitative data to explore participants’ experiences regarding online versus in-person delivery. Finally, as is often the case in family research,^14^ there was a low number of male carers in the current study.

Services offering E-PAtS to parents could determine the mode of delivery on the basis of resource availability, reach and parent preference. Offering choice and inviting families to attend through the mode that is right for them is therefore recommended for services, along with ongoing efforts to increase engagement for male carers. In addition to further examination of the effectiveness of E-PAtS in relation to its hypothesised primary outcome (parental mental well-being), future non-inferiority trials need to examine whether E-PAtS groups delivered online are equally effective as those delivered face to face.

## Data Availability

The data that support the findings of this study are available from the first author (H.N.) on reasonable request.

## About the authors

H.N., Graduate Mental Health Support Worker, Llygad Early Years Experience Team, Cardiff and Vale University Health Board, Cardiff, UK. E.J., Clinical Psychologist and Service Lead, Llygad Early Years Experience Team, Cardiff and Vale University Health Board, Cardiff, UK. N.G., Senior Researcher Fellow, Intellectual Disabilities Research Institute (IDRIS), University of Birmingham, Birmingham, UK. C.A.W., Post-Doctoral Researcher, Division of Psychiatry, University College London, London, UK and School of Psychology, University of Birmingham, Birmingham, UK. V.T., Professor of Neurodevelopmental Research, Division of Psychiatry, University College London, London, UK and Tavistock & Portman NHS Foundation Trust, London, UK. J.B., Professor of Communication, Health and Social Care, Intellectual Disabilities Research Institute (IDRIS), University of Birmingham, Birmingham, UK.

## Table 1 Long description

The table presents demographic characteristics of participants receiving Early Positive Approaches to Support. It has 10 rows and 4 columns. The columns are Demographic characteristics, Age (years), Gender, n (%), and Caregiver type, n (%). The rows are Age (years), Gender, n (%), and Caregiver type, n (%). The table includes the following data: Age (years): Mean (s.d.): 34.56 (6.86), Range: 24-53, Missing information: 43 (43). Gender, n (%): Female: 55 (55), Male: 3 (3), Missing information: 42 (42). Caregiver type, n (%): Primary carer: 74 (74), Secondary carer: 3 (3), Missing information: 23 (23). Ethnicity, n (%): White British: 23 (23), White other: 8 (8), African: 2 (2), Indian: 1 (1), Asian: 5 (5), Asian other: 2 (2), Black other: 2 (2), Multiple ethnic: 3 (3), Any other ethnic group: 1 (1), Missing information: 53 (53).

Navigate back to Table 1.

## Table 2 Long description

A table with four rows and four columns comparing pre- and post-intervention outcomes and effect sizes. The columns are labeled Outcome, Pre-intervention (95% CI), Post-intervention (95% CI), and Cohen's d (95% CI). The rows are labeled E-PAtS programme evaluation, Warwick-Edinburgh Mental Well Being Scale, Brief Parental Self-Efficacy Scale, and Sheffield Learning Disability Outcome Measure. Row 1: E-PAtS programme evaluation, Pre-intervention (34.21 (33.00, 35.42)), Post-intervention (42.23 (41.05, 43.41)), Cohen's d (1.28 (0.97, 1.59)). Row 2: Warwick-Edinburgh Mental Well Being Scale, Pre-intervention (42.41 (40.43, 44.39)), Post-intervention (49.97 (47.06, 52.88)), Cohen's d (0.47 (0.30, 0.63)). Row 3: Brief Parental Self-Efficacy Scale, Pre-intervention (18.04 (17.11, 18.98)), Post-intervention (22.48 (21.14, 23.81)), Cohen's d (0.73 (0.47, 0.99)). Row 4: Sheffield Learning Disability Outcome Measure, Pre-intervention (26.16 (25.08, 27.25)), Post-intervention (29.08 (27.20, 30.97)), Cohen's d (0.34 (0.11, 0.56)).

Navigate back to Table 2.

## Table 3 Long description

A table comparing outcomes of online versus in-person delivery methods for a program, including effect sizes. The table has four rows and four columns. The columns are labeled Outcome, Online (95% CI), In-person (95% CI), and Cohen's d (95% CI). The rows are labeled with different outcomes: E-PAtS programme evaluation, Warwick-Edinburgh Mental Well Being Scale, Brief Parental Self-Efficacy Scale, and Sheffield Learning Disability Outcome Measure. Each row contains values for the respective outcomes under the columns Online (95% CI), In-person (95% CI), and Cohen's d (95% CI).

Navigate back to Table 3.
